# Effects of Seaweed Polysaccharide on the Growth and Physiological Health of Largemouth Bass, *Micropterus salmoides*

**DOI:** 10.3390/antiox14010052

**Published:** 2025-01-04

**Authors:** Dongyu Huang, Jiaze Gu, Hualiang Liang, Mingchun Ren, Chunyu Xue

**Affiliations:** 1 Key Laboratory of Integrated Rice-Fish Farming Ecology, Ministry of Agriculture and Rural Affairs, Freshwater Fisheries Research Center, Chinese Academy of Fishery Sciences, Wuxi 214081, China; 2 Wuxi Fisheries College, Nanjing Agricultural University, Wuxi 214081, China

**Keywords:** seaweed polysaccharide, immunity, endoplasmic reticulum stress

## Abstract

A seven-week trial was designed to evaluate the effects of dietary seaweed polysaccharide (SP) supplementation on the growth performance and physiological health of largemouth bass. The results reveal that the 0.05SP group showed the best growth performance. The mRNA expression levels of *tor*, *4ebp1*, and *igf1* genes were remarkably down-regulated in the 0.15SP and 0.2SP groups compared to the control group. The CAT activities were significantly increased in the 0.05SP and 0.1SP groups, and the GSH-Px activity was increased in the 0.15SP group. The expression of the immune response-related gene *nfκb* was significantly down-regulated in the 0.1SP group, and those of *tnfα* and *il-8* were at the maximum in the control group. Moreover, the expression of *il-10* in the 0.15SP and 0.2SP groups was significantly down-regulated. Furthermore, endoplasmic reticulum stress (ERS)-related expression of *atf6* was the highest in the control group. Furthermore, the *chopα* and *bax* expression levels in the 0.15SP and 0.2SP groups were significantly down-regulated compared with other groups. In addition, the highest expression level of *bcl-xl* was observed in the 0.15SP group. Finally, the quadratic regression analysis of antioxidant, immune, and ERS core parameters (CAT, *nf-κb*, and *bcl-xl*) determined 0.06–0.11% to be the optimal SP supplemental level in largemouth bass diets.

## 1. Introduction

Largemouth bass (*Micropterus salmoides*) or California bass is popular amongst consumers and anglers due to its tender and flavorful meat. It is a bestseller and also known as “freshwater grouper” in the international market [1]. Largemouth bass production in 2023 has been estimated at over 888,000 tonnes [2] and it plays an indispensable role in securing the supply of high-quality protein sources for human beings. So far, the compound feed for largemouth bass has been successfully developed and widely used. However, the rising price of fishmeal in recent years has led to an increase in the costs of these feeds, thereby limiting the growth and economic benefits of the largemouth bass industry, farmers, and enterprises.

Therefore, fishmeal replacements have been extensively investigated by researchers. Notably, enzymatically digested or fermented plant proteins, insect proteins, and single-cell proteins have shown good results [3,4,5]. In addition, composite protein sources from some plants and animals (e.g., chicken, soybean, shrimp, and blood meals) [6,7] can replace fishmeal partially in largemouth bass diets without affecting their growth performance. However, replacing high proportions of fishmeal often results in significantly reduced growth performance, impaired immunity, and exacerbation of metabolic diseases in largemouth bass [8,9]. Therefore, the development and use of low-fishmeal diets should focus on replacing fishmeal with other protein sources without affecting growth performance while improving the immunity of largemouth bass.

In recent years, several additives have been gradually introduced into low-fishmeal feeds without showing negative effects on feeding, metabolism, and immunity [10,11,12]. Polysaccharides are functional additives that show diverse immunomodulatory effects including bactericidal, antiviral, and anti-tumor effects, and are considered novel types of immune enhancers in the aquaculture industry [13,14,15]. Specifically, seaweed polysaccharide (SP) is a class of macromolecular compounds extracted from marine algae with good biocompatibility, degradability, diversified activities, and low toxicity. Their application in aquatic animal feeds has been widely recognized, and they can regulate the immune system of fish [16]. Previous studies demonstrated that dietary SP supplementation improved growth, immune response, and disease resistance in many aquatic animals, and the addition of 1 g/kg SP to the diet improved the growth performance, enhanced non-specific immunity, and regulated intestinal function in banana shrimp (*Fenneropenaeus merguiensis*) [17]. Diets supplemented with 500 mg/kg SP had the greatest growth-promoting effect on Giant freshwater prawn (*Macrobrachium rosenbergii*), as observed from the immune response, and the 100 mg/kg concentration of SP significantly increased their survival rate [18]. In addition, the addition of SP (200 or 300 mg/kg diet) is important for improving growth, antioxidant immune status, and inflammatory response in rohu carp (*Labeo rohita*) during *Flavobacterium columnare* infection [19]. And 0.15g/kg dietary SP supplementation could significantly improve the growth performance of rabbitfish (*Siganus canaliculatus*), whereas the addition of 0.10g/kg SP improved the feed utilization efficiency (reduced feed conversion and increased protein utilization) and significantly reduced cumulative mortality following challenges from *Vibrio parahaemolyticus* [20]. However, the application of SP in largemouth bass diets has not been reported. Moreover, despite its popularity as an additive in aquafeed, its ability to alleviate the negative effects of low-fishmeal feeds needs further investigation.

Therefore, the present study investigates the effects of dietary SP supplementation on the growth and physiological health of largemouth bass to provide research references and guidance on the application of SP in aquaculture.

## 2. Materials and Methods

### 2.1. Experimental Diets and Additives

Based on previous studies of seaweed polysaccharides as additives in some fish species, which found that the optimal levels of SP addition generally focused on the range of 0.01–0.15% [19,20,21,22], as well as the recommended dosage value of this product in aquatic animals of 0.05–0.2% (Hubei Jingruitianheng Biotechnology Co., Ltd., Yichang, China), the designed addition levels for this experiment were 0%, 0.05%, 0.1%, 0.15%, and 0.2%. The basic formula was shown in Table 1. Initially, various raw materials were crushed and passed through an 80-mesh sieve and then weighed according to the feed formula. Firstly, mix lysine, methionine, vitamin and mineral premix, mono-calcium phosphate, choline chloride, and other traces or small amounts of ingredients, then add fish meal, domestic poultry by product meal, soybean meal, soya protein concentrate, wheat gluten, and other large amounts of ingredients step by step. Five groups of ingredients were weighed, which were labeled as control, 0.05SP, 0.1SP, 0.15SP, and 0.2SP, respectively. Since 10 kg diets were made for each group, 0 g, 5 g, 10 g, 15 g, and 20 g SP were added to each of the five groups based on SP addition levels of 0%, 0.05%, 0.1%, 0.15%, and 0.2%. And then add fish oil, soybean oil, and the appropriate amount of water by mixing. Finally, add an appropriate volume of water with the extruder made of 1.0 mm extruded feed and dry it for use.

### 2.2. Experimental Fish

Fish species were provided by Chia Tai Aquatic Products (Huzhou) Co., Ltd. (Huzhou, China). Healthy and uniformly sized largemouth bass weighing approximately 1.52 ± 0.01 g were divided into five groups: control, 0.05% SP (0.05SP), 0.1% SP (0.1SP), 0.15% SP (0.15SP), and 0.2% SP (0.2SP). The experiment was performed in triplicates for all groups with 20 fish each, and a total of 18 floating cages were set up outdoors for this purpose.

### 2.3. Experimental Management

The experimental fish were temporarily reared in outdoor floating cages at the Freshwater Fisheries Research Center (FFRC) of the Chinese Academy of Fisheries Sciences for seven days. Thereafter, the fish were fed with the experimental diets twice daily until apparent satiety. The parameters (temperature 26 ± 2 °C, pH 7.2–7.8, and dissolved oxygen 6.5–7.6 mg/L) were maintained to ensure the standard of water quality.

### 2.4. Sample Collection

After seven weeks of experimental rearing, the fish were fasted for 24 h, followed by measuring the total weight of the fish in each cage to calculate the growth performance. Next, six fish were randomly selected from each cage and immediately anesthetized by putting them into MS-222 (100 mg/L). The liver tissues from three fish were collected and preserved in 4% paraformaldehyde for further histopathological observation. Simultaneously, the liver tissues from the remaining three fish were collected into freezing tubes and stored at −80 °C for subsequent liver enzyme activity and gene expression analysis. Moreover, the remaining fish from each cage were stored in sealed bags in a refrigerator at −20 °C for routine whole fish nutrient analysis.

### 2.5. Sample Analysis

The nutrient compositions of experimental diets and whole fish were determined according to the methods of AOAC [23], the dry matter content was calculated by drying at atmospheric pressure in an oven at 105 °C until constant weight, the crude protein content of the samples was determined by Kjeldahl nitrogen determination, the crude lipid content was determined by Soxhlet extraction using ether lipids, and the ash content was calculated by burning at 560 °C for 5 h in a muffle furnace. And the liver antioxidant parameters were estimated using a standard kit. Detailed information is displayed in Table 2.

The histopathological examination utilized the tissue samples after 4% paraformaldehyde fixation. Thereafter, liver tissues of juvenile largemouth bass were fixed by using 4% paraformaldehyde for more than 48 h. The tissue was then used as a reference for the fixation of the liver tissue. Samples were dehydrated in a dehydrator (Leica, TP1020, Wetzlar, Germany) with an alcohol gradient and treated with wax immersion. The wax-impregnated tissues were embedded in an embedding machine (Leica, EG1150), cooled on a freezing table at −20 °C, and then sectioned to a thickness of 4 μm on a paraffin slicer (Leica, RM2235). After HE staining, hematoxylin stained the nuclei and eosin stained the cytoplasm. Finally, the samples were dehydrated, sealed, and examined with a microscope (Nikon, H500S, Tokyo, Japan) and images were captured and analyzed [24].

For the gene expression analysis, total RNA was extracted from largemouth bass liver tissue using the RNAiso plus kit (Takara, Dalian, China) as described in the kit instructions. The concentration and purity of total RNA were determined using a Nano drop 2000 spectrophotometer at 260 nm and 280 nm to ensure that the OD260/OD280 values of the samples were between 1.8 and 2.0, and all the samples were diluted uniformly with RNA-free DEPC water to 60 ng/μL for testing. Samples to be tested were prepared using HiScript^®^ II One Step qRT-PCR SYBR Green Kit (Q221-01, Vazyme, Nanjing, China) reagent, and then the RT-qPCR reaction was carried out on a CFX96 Touch (Bio-Rad, Hercules, CA, USA). One Step qRT-PCR reaction conditions were as follows: 50 °C, 3 min; 95 °C, 30 s; 95 °C, 10 s; 60 °C, 30 s, 40 cycles; 95 °C, 15 s; 60 °C, 60 s; and 95 °C, 15 s. The primers were designed using the NCBI Primer-BLAST with *β-actin* as the reference gene (Table 3). Finally, the expression levels of each target gene were calculated using the 2^−ΔΔCT^ method [25].

### 2.6. Statistical Analysis

The data were analyzed using the SPSS25.0 software and expressed as mean ± standard error. The data were first tested for normal distribution and were then subject to a chi-square analysis. In the case of normal distribution and homogeneity of variance, one-way ANOVA was used, followed by Duncan’s test for multiple comparisons of significant differences.

## 3. Results

### 3.1. Growth Performance

The final weight (FW), weight gain rate (WGR), and specific growth rate (SGR) decreased significantly in a non-linear way (*p* < 0.05) with an increase in the dietary SP concentration (Table 4). The highest values of FBW, WGR, and SGR were observed for the 0.05SP group, which were significantly higher than the 0.2SP group (*p* < 0.05), whereas the 0.05SP group showed the lowest feed conversion ratio (FCR; *p* < 0.05). In addition, the survival rate (SR) was unaffected among all groups (*p* > 0.05).

### 3.2. Whole-Body Composition

As shown in Table 5, the moisture, crude protein, crude lipid, and crude ash contents were not significantly different between the groups (*p* > 0.05).

### 3.3. Antioxidant Parameters

Table 6 presents the liver antioxidant parameters. Briefly, the CAT activity increased in a parabolic fashion with increasing dietary SP contents (*p* < 0.05). Specifically, compared with the control group, the CAT activities in the 0.05SP and 0.1SP groups were significantly increased (*p* < 0.05). Moreover, the GSH-Px activity increased significantly in the 0.15SP group compared with the control group (*p* < 0.05). In addition, the SOD activities and GSH, T-AOC, and MDA contents remained unaffected by dietary SP levels (*p* > 0.05).

### 3.4. Histopathological Analysis

**Figure 1:**
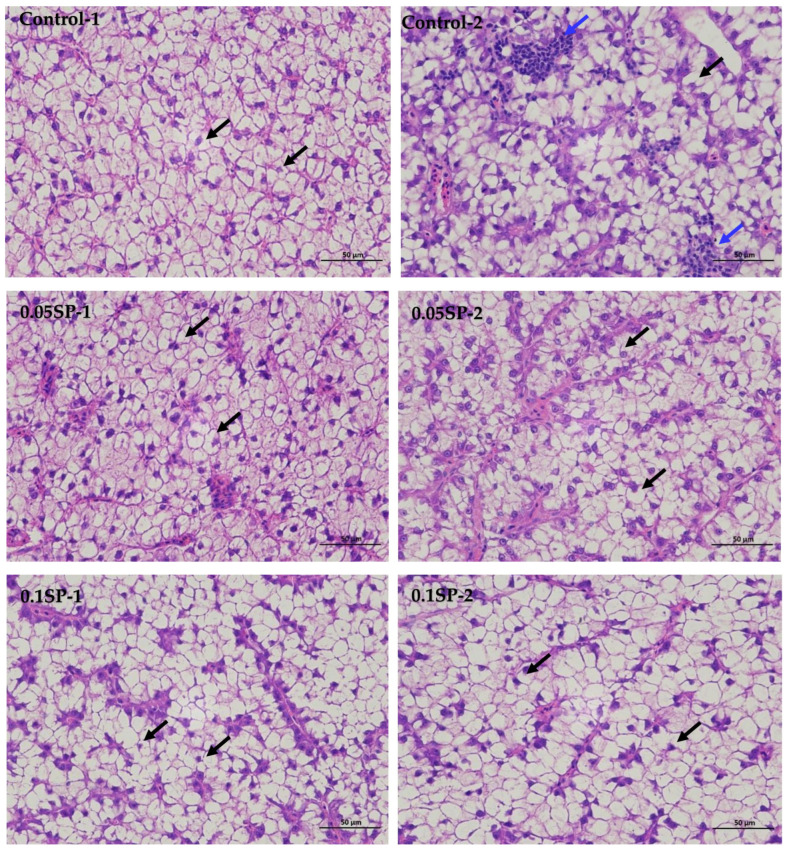

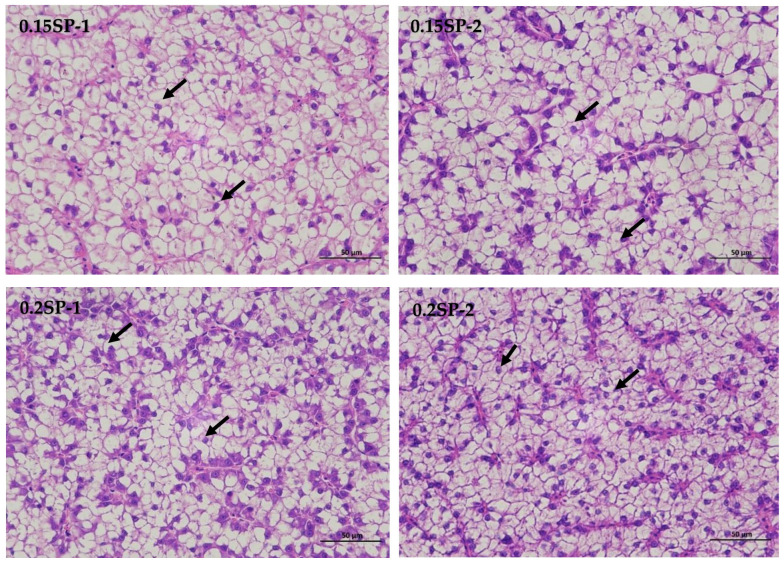
Liver sections of largemouth bass stained with Hematoxylin and Eosin (HE) at different SP supplementation levels (400× magnification). Black arrows indicate a vacuolated cytoplasm and blue arrows highlight small focal infiltrates of lymphocytes.

The results show that, in the liver tissues of the group supplemented by 0.05%SP–0.2%SP, the hepatocytes were arranged in two rows, forming a plate-like morphology and scattered sequentially around the central vein. Precisely, the hepatocytes were irregularly polygonal with nuclei centered or crowded on one side. In addition, the cytoplasm was vacuolated (black arrows), and there was no significant dilatation of hepatic blood sinusoids between the plates, and there was no necrotic or inflammatory cell infiltration. However, in the control group (Control-2), a few small focal infiltrations of lymphocytes were occasionally seen (blue arrows) (Figure 1).

### 3.5. Expression of Protein Metabolism-Related Genes

**Figure 2:**
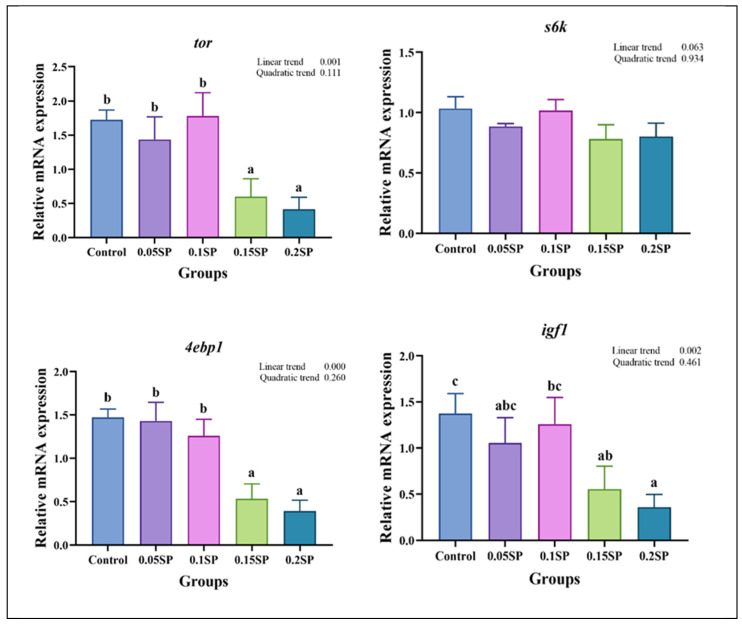
Relative gene expressions of protein metabolisms with different SP levels. Superscripts of different letters (a, b, c) indicate significant differences between groups (*p* < 0.05).

Figure 2 shows the expression of protein metabolism-related genes in response to SP supplementation. The expression of *tor* decreased with an increase in SP contents (*p* < 0.05). Specifically, the *tor* mRNA levels in the 0.05SP and 0.1SP groups were the same as that of the control group (*p* > 0.05) and significantly higher relative to the 0.15SP and 0.2SP groups (*p* < 0.05). In addition, the *4ebp1* and *igf1* expressions were similar to that of *tor* and were significantly down-regulated in the 0.15SP and 0.2SP groups compared to the control, 0.05 SP, and 0.1 SP groups (*p* < 0.05).

### 3.6. Expression of Immune Response-Related Genes

**Figure 3:**
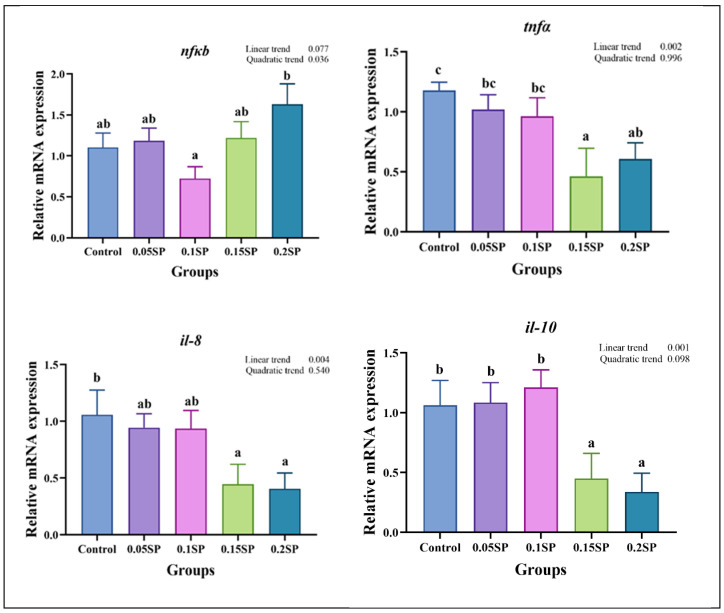
Relative gene expressions of immune responses with different SP levels. Superscripts of different letters (a, b, c) indicate significant differences between groups (*p* < 0.05).

Figure 3 presents the expression of immune response-related genes. Briefly, the *nfκb* expression decreased in a parabolic fashion with increasing dietary SP contents (*p* < 0.05), with the least expression observed in the 0.1SP group, which, in turn, was lower than the 0.2SP group (*p* < 0.05). In addition, the expression of *tnfα*, *il-10*, and *il-8* decreased linearly with increasing SP contents, and lower expressions of *tnfα* and *il-8* were observed for the 0.15SP and 0.2SP groups relative to the control group (*p* < 0.05). Similarly, the *il-10* expression levels were significantly down-regulated in the 0.15SP and 0.2SP groups (*p* < 0.05).

### 3.7. Expression of Endoplasmic Reticulum Stress-Related Genes

**Figure 4:**
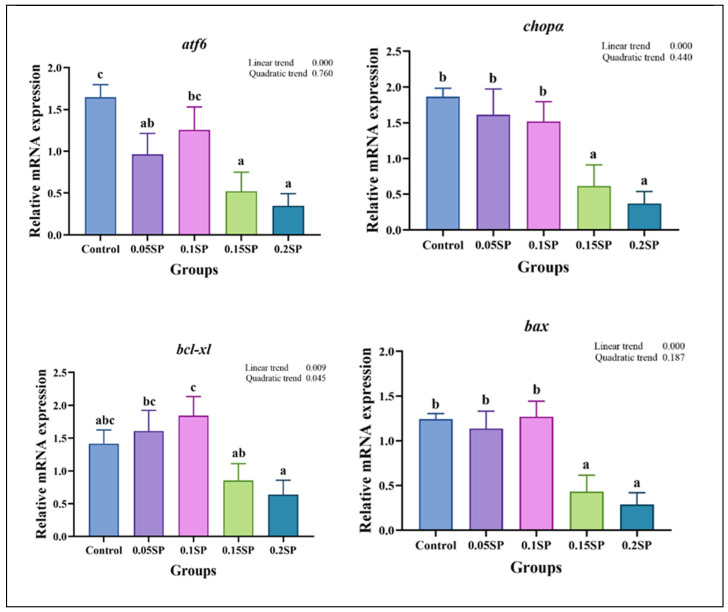
The relative gene expressions of endoplasmic reticulum stress with different SP levels. Superscripts of different letters (a, b, c) indicate significant differences between groups (*p* < 0.05).

The results demonstrate a decreased expression of *atf6*, *chopα*, *bcl-xl*, and *bax* genes linearly with increasing dietary SP contents (*p* < 0.05; Figure 4). Out of all the groups, the expression of *atf6* was significantly higher in the control group (*p* < 0.05). In addition, compared with the other groups, the expression levels of *chopα* and *bax* in the 0.15SP and 0.2SP groups were significantly down-regulated (*p* < 0.05). In addition, the *bcl-xl* expression was up-regulated in a parabolic fashion with increasing dietary SP contents (*p* < 0.05). Similarly, the highest expression level of *bcl-xl* was observed for the 0.1SP group, which was higher than that for the 0.15SP and 0.2SP groups (*p* < 0.05).

### 3.8. Regression Analysis

**Figure 5:**
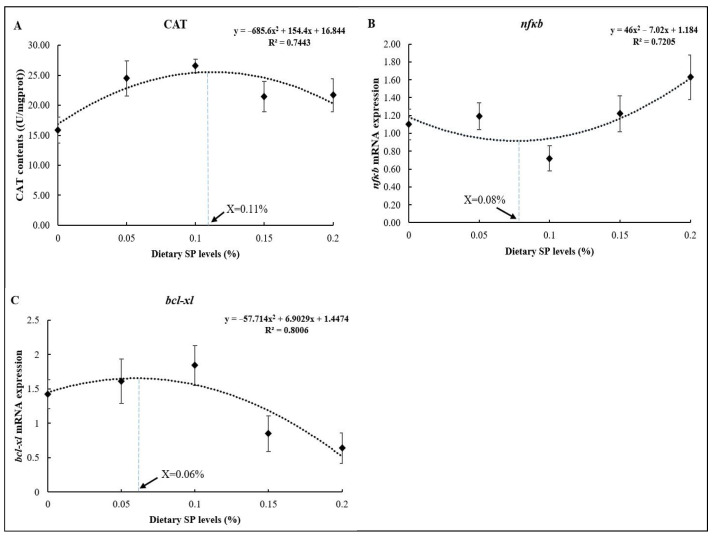
Regression analysis between dietary SP levels and different parameters ((**A**) CAT; (**B**) *nfκb*; (**C**) *bcl-xl*).

As shown in Figure 5, the optimum dietary SP levels based on the CAT, *nfκb*, and *bcl-xl* results were 0.11% (Figure 5A), 0.08% (Figure 5B), and 0.06% (Figure 5C) of the diets, respectively, as determined using the quadratic regression analysis.

## 4. Discussion

Our results suggest that 0.05% SP addition to the largemouth bass diet increased the FW, WGR, and SGR and reduced the FCR non-significantly relative to the control group. In addition, increasing the SP content did not promote the growth performance, but rather decreased it, thereby suggesting that a moderate amount of SP had a positive but non-significant effect on the growth performance. As reported, kelp and purslane polysaccharides improved the growth performance and enhanced the immunity of juvenile yellow croaker (*Larimichthys crocea*); however, the improvement in growth performance was not positively proportional to the amount of polysaccharide added [28]. Reportedly, polysaccharides are not directly involved in nutrient metabolisms, and their growth-promoting effects may be closely related to their immunomodulatory effects [29,30]. In addition, TOR regulates the cell cycle and growth signaling and plays a critical role in protein synthesis [31]. It can regulate cell growth, proliferation, apoptosis, and autophagy by activating downstream 4EBP1 and S6K1 [32]. Compared to the control group, no change was shown in *tor* and *4ebp1* mRNA expression in the 0.05SP and 0.1SP groups, whereas it was down-regulated in the 0.15SP and 0.2SP groups. In addition, *igf1* mRNA expression was similar to that of the TOR signaling pathway, which in turn regulates animal growth and development [33]. These results suggest that a moderate amount of SP (0.05–0.1%) did not influence the growth performance of largemouth bass, whereas excessive amounts (0.15–0.2%) reduced it. Reportedly, appropriate doses of polysaccharides can play a positive role in immunomodulation to promote growth. In contrast, higher levels of polysaccharides led to the diversion of digestible energy and protein towards immune function, thereby reducing growth performance [30]. Therefore, the specific mechanisms underlying the action of SP on the growth performance need further investigation.

Furthermore, some polysaccharides do not interfere with fish composition, including the effects of SP on yellow croaker [25] and *Lycium barbarum* polysaccharide on juvenile golden pompano (*Trachinotus ovatus*) [29]. Conversely, some studies reported that dietary garlic (*Allium sativum*) crude polysaccharides increased crude protein and decreased crude lipid and ash contents of African catfish (*Clarias gariepinus*) [34]. Similarly, β-glucomannan increased protein and decreased crude lipid contents in largemouth bass [35]. These differences may be attributed to the different structures and active components of polysaccharides.

SP can scavenge free radicals in animal bodies, thereby improving their antioxidant capacity and alleviating oxidative damage [36]. In this study, SP supplementation did not cause damage to the liver tissue of largemouth bass. As one of the biologically active substances of seaweeds, SP has diverse physiological functions, such as immune regulation, antioxidant, and liver protection, along with others [37]. Based on the histopathological analysis in this experiment, it was found that the infiltration of inflammatory cells was occasionally found in the liver tissue of largemouth bass in the control group, probably due to the low fishmeal diets, which may lead to liver damage and inflammation [38]. In addition, dietary SP supplementation is effective in reducing inflammation and avoiding liver damage, mainly by enhancing the activity of antioxidant enzymes to enhance free radical scavenging [16] and by modulating relevant signaling pathways to reduce inflammation [39]. Furthermore, our results demonstrate that a moderate amount of SP increased the CAT and GSH-Px activities, with the highest activity found in the 0.01SP and 0.15SP groups, respectively, without affecting the MDA content. According to a previous study, many natural polysaccharides are effective free radical scavengers due to their potent antioxidant activity [40]. Similar effects of SP are mainly indicated by the increased activities of CAT, SOD, etc., and by scavenging hydroxyl radicals and superoxide radicals [41,42]. Thus, this experiment indicated that dietary supplementation with 0.05–0.15% SP could increase the antioxidant enzymatic activities in largemouth bass and reduce oxidative damage.

SP is also associated with the regulation of immune system function by modulating the NF-κB signaling pathway to regulate the expression of immune-related molecules [43]. A previous study reported that polysaccharides from *Saccharina japonica* inhibit lipopolysaccharide-induced activation of the NF-κB pathway and act as anti-inflammatory agents [44]. Wang et al. [45] also reported that lambda polysaccharides could suppress the NF-κB signaling pathway, leading to the reduced expression of inflammatory factors in LPS-induced RAW264.7 inflammatory cells. Our study also shows that the expression of *nf-κb* decreased in a parabolic fashion with increasing dietary SP contents, and the lowest expression was found in the 0.1SP group. Therefore, SP could also restore the imbalanced expression of inflammatory factors downstream of NF-κB, thereby regulating the body’s immune system. Moreover, SP reduced the *il-8* and *tnf-α* expression in a mouse model with LPS-induced vascular endothelial cell inflammation. In addition, it effectively decreased the NO production in the pathological state while increasing it in the physiological state [46]. Tian and Liu [47] showed that purslane polysaccharides inhibited *tnf-α* expression in enterovirus 71-inoculated mice and promoted *il-10* expression, thus maintaining the dynamic balance of cytokine levels in the organism. In our study, dietary SP supplementation down-regulated the *tnf-α* and *il-8* expression levels, whereas no significant differences in the expression of *il-10* in the 0.05SP and 0.1SP groups were observed relative to the control group. Collectively, these results suggest that 0.05–0.1% dietary SP supplementation could inhibit the expression of inflammatory factors to some extent and improve the immunity of largemouth bass.

In addition, endoplasmic reticulum stress (ERS) is a cellular stress response caused by an abnormal accumulation of proteins, leading to apoptosis and other adverse reactions in the animal’s body [48]. The ATF6/CHOP is an apoptosis-related pathway [49], where CHOP, a key signaling molecule, activates apoptosis in the endoplasmic reticulum [50,51]. In this study, the expressions of *chopα* and its upstream regulator *atf6* were down-regulated by dietary SP supplementation. Previous studies have also shown that Astragalus polysaccharide could inhibit the ATF6/CHOP pathway, thereby inhibiting mucosal apoptosis by decreasing the Bax expression [52]. When ERS fails to resolve, apoptosis can also be inhibited by increasing the expression of Bcl-xl [53]. Our results also demonstrate that *bax* mRNA expression down-regulated linearly with increasing dietary SP contents, whereas the *bcl-xl* mRNA expression was up-regulated in the 0.1SP group. Therefore, appropriate dietary SP supplementation could effectively alleviate ERS and apoptosis by inhibiting the ATF6/CHOP signaling pathway. However, the mechanisms underlying these effects on aquatic animals through ERS are partly understood and need further research.

## 5. Conclusions

Overall, 0.05% SP supplementation could improve the growth performance of largemouth bass, and appropriate SP supplementation (0.05–0.1%) maintains liver health by increasing the antioxidant enzymatic activity, regulating the NF-κB signaling pathway to improve immunity, and inhibiting the ATF6/CHOP signaling pathway to alleviate ERS. Moreover, the optimum dietary SP levels were 0.11%, 0.08%, and 0.06% of the diet based on the CAT, *nfκb*, and *bcl-xl*, respectively, as determined using quadratic regression analysis. However, due to the lack of precise and sufficient in vivo evidence of SP and the uncertainty of the conformational relationship, the application of the mechanism is not clear in bottleneck scenarios, and so SP is not widely used; further research on the economic value of SP has a long way to go, and it is expected that with the continuous and in-depth study of SP, it will be better used in the field of aquatic products.

## Figures and Tables

**Table 1:** Basic formula (% dry matter).

Ingredients			
Fish meal	25.00	Fish oil	2.80
Domestic poultry by product meal	12.00	Soybean oil	5.00
Soya protein concentrate	12.57	Mono-calcium Phosphate	3.08
Soybean meal	15.77	Mineral and Vitamin premix	2.00
Wheat gluten	2.90	Choline Chloride	0.50
Porcine hemoglobin meal	5.12	Vitamin C	0.10
Wheat flour	8.00	Lysine	0.03
Tapioca starch	5.00	Methionine	0.13
Total	100.0		
Analyzed proximate composition			
Crude protein (%)	47.37 ± 0.08		
Crude lipid (%)	11.61 ± 0.15		

Above ingredients were obtained from Wuxi Tongwei BioMar feedstuffs Co., Ltd. (Wuxi, China).

**Table 2:** The chemical analyses used in the experiment.

Items	Methods	Assay Kits/Testing Equipment
Composition of diets/ingredients		
Moisture	Drying method	Electric blast drying oven (Shanghai Yiheng Scientific Instrument Co., Ltd., Shanghai, China)
Protein	Kjeldahl	Auto kieldahl apparatus: Hanon K1100 (Jinan Hanon Instruments Co., Ltd., Jinan, China)
Lipid	Soxhlet	Auto fat analyzer: Hanon SOX606 (Jinan Hanon Instruments Co., Ltd., Jinan, China)
Ash	Combustion	Muffle: XL- 2A (Hangzhou Zhuochi Instrument, Hangzhou, China)
Plasma parameters related to antioxidant capacity		
SOD ^1^	WST-1 method	Assay kits purchased from Jian Cheng Bioengineering Institute (Nanjing, China); Spectrophotometer (Thermo Fisher Multiskan GO, Shanghai, China).
T-AOC ^2^	ABTS method	
GSH ^3^	Microplate method	
GSH-Px ^4^	Colorimetric method	
MDA ^5^	TBA method	
CAT ^6^	Ammonium molybdenum acid method	

^1^ SOD, Superoxide dismutase; ^2^ T-AOC, Total antioxidant capacity; ^3^ GSH, Glutathione; ^4^ GSH-Px, Glutathione peroxidase; ^5^ MDA, Malondialdehyde; ^6^ CAT, Catalase.

**Table 3:** The specific primers for the reference gene and target genes.

Genes		Primer Sequence (5′-3′)	Accession Number/Reference
*tor*	Forward	TTTGGAACCAAACCCCGTCA	XM_038723321.1
	Reverse	ATCAGCTCACGGCAGTATCG	
*s6k*	Forward	TCCAGAGACTCGTGACACCT	XM_038713349.1
	Reverse	AGCTTGGCATACTCTGAGGC	
*4ebp1*	Forward	CCAGGATCATCTATGACCGAAAG	XM_038703879.1
	Reverse	TGCAGCGATATTGTTGTTGTTC	
*igf1*	Forward	CCTCTGCCTGTGTATAATCA	XM_038738328.1
	Reverse	TGTCCGTCTTAGCCATCT	
*nfκb*	Forward	AGAAGACGACTCGGGGATGA	XM_038699793.1
	Reverse	GCTTCTGCAGGTTCTGGTCT	
*tnfα*	Forward	CTTCGTCTACAGCCAGGCATCG	XM_038710731.1
	Reverse	TTTGGCACACCGACCTCACC	
*il-8*	Forward	GAGGGTACATGTCTGGGGGA	XM_038713529.1
	Reverse	CCTTGAAGGTTTGTTCTTCATCGT	
*il-10*	Forward	CGGCACAGAAATCCCAGAGC	XM_038696252.1
	Reverse	CAGCAGGCTCACAAAATAAACATCT	
*atf6*	Forward	CACCTCATAACACCTACAGT	XM_038716053.1
	Reverse	GCAACACCACAGACATCT	
*chopα*	Forward	AGAGGACAGCAGCAGTAA	XM_038701049.1
	Reverse	GAGCGATGATGAGCAGAT	
*bcl-xl*	Forward	CATCCTCCTTGGCTCTGG	[26]
	Reverse	GGGTCTGTTTGCCTTTGG	
*bax*	Forward	ACTTTGGATTACCTGCGGGA	[27]
	Reverse	TGCCAGAAATCAGGAGCAGA	
*β-actin*	Forward	GGTGTGATGGTTGGTATGG	MH018565.1
	Reverse	CTCGTTGTAGAAGGTGTGAT	

Note: *tor*, target of rapamycin; *s6k*, ribosomaiprotein S6 kinase; *4ebp1*, 4E binding protein 1; *igf1*, insulin-like growth factor 1; *nfκb*, nuclear factor kappa-B; *tnfα*, tumor necrosis factor-α; *il-8*, interleukin 8; *il-10*, interleukin 10; *atf6*, activating transcription factor 6; *chopα*, clonal hematopoiesis of indeterminate potential α; *bcl-xl*, B-cell lymphoma-xl; *bax*, bax protein.

**Table 4:** Effects of seaweed polysaccharide on growth performance of largemouth bass.

Groups	IW (g)	FW (g)	WGR (%)	SGR (%/Day)	FCR	SR (%)
Control	1.51 ± 0.01	7.38 ± 0.08 ^ab^	389.02 ± 6.74 ^b^	3.69 ± 0.10 ^b^	0.81 ± 0.01 ^ab^	100.00 ± 0.00
0.05SP	1.53 ± 0.01	7.48 ± 0.15 ^b^	389.46 ± 7.79 ^b^	3.70 ± 0.04 ^b^	0.79 ± 0.03 ^a^	100.00 ± 0.00
0.1SP	1.52 ± 0.01	7.23 ± 0.07 ^ab^	375.99 ± 5.16 ^ab^	3.63 ± 0.03 ^ab^	0.82 ± 0.01 ^ab^	100.00 ± 0.00
0.15SP	1.52 ± 0.01	7.22 ± 0.16 ^ab^	374.92 ± 12.62 ^ab^	3.62 ± 0.06 ^ab^	0.83 ± 0.02 ^ab^	100.00 ± 0.00
0.2SP	1.51 ± 0.01	7.01 ± 0.03 ^a^	360.93 ± 3.54 ^a^	3.55 ± 0.02 ^a^	0.85 ± 0.01 ^b^	100.00 ± 0.00
*p*-value						
Linear trend	0.471	0.018	0.017	0.015	0.050	—
Quadratic trend	0.540	0.398	0.586	0.536	0.357	—

Note: Superscripts of different letters (a and b) in the same column indicate significant differences between groups (*p* < 0.05). WGR (%) = 100 × (FW (g) − IW (g))/IW (g). SGR (% day^−1^) = 100 × [(ln (FW(g)) − ln (IW (g)))/days]. FCR = dry feed fed (g)/(FW (g) − IW (g)). SR (%) = 100 × (survival fish number/total fish number).

**Table 5:** Effects of seaweed polysaccharide on body compositions of largemouth bass.

Groups	Moisture (%)	Crude Protein (%)	Crude Lipid (%)	Crude Ash (%)
Control	75.87 ± 0.66	14.53 ± 0.13	5.67 ± 0.89	3.01 ± 0.14
0.05SP	76.71 ± 0.17	14.61 ± 0.11	5.28 ± 0.28	3.06 ± 0.05
0.1SP	76.83 ± 0.38	14.14 ± 0.30	5.79 ± 0.32	2.89 ± 0.09
0.15SP	76.53 ± 0.53	14.13 ± 0.22	5.69 ± 0.46	3.05 ± 0.11
0.2SP	76.27 ± 0.54	14.18 ± 0.11	6.54 ± 0.51	3.08 ± 0.13
*p*-value				
Linear trend	0.692	0.077	0.234	0.703
Quadratic trend	0.176	0.582	0.373	0.472

**Table 6:** Effects of seaweed polysaccharide on liver antioxidant parameters of largemouth bass.

Groups	CAT (U/mgprot)	SOD (U/mgprot)	GSH (μmol/gprot)	GSH-Px (U/mgprot)	T-AOC (mmol/gprot)	MDA (nmol/mgprot)
Control	15.84 ± 2.17 ^a^	7.09 ± 0.23	0.26 ± 0.09	12.19 ± 1.29 ^a^	0.25 ± 0.03	0.48 ± 0.15
0.05SP	24.49 ± 2.92 ^b^	6.92 ± 0.29	0.22 ± 0.04	13.73 ± 1.26 ^ab^	0.24 ± 0.03	0.47 ± 0.08
0.1SP	26.55 ± 1.10 ^b^	6.44 ± 0.16	0.24 ± 0.09	12.98 ± 0.72 ^ab^	0.27 ± 0.03	0.46 ± 0.07
0.15SP	21.44 ± 2.54 ^ab^	7.03 ± 0.37	0.21 ± 0.06	16.19 ± 0.77 ^b^	0.23 ± 0.05	0.57 ± 0.05
0.2SP	21.68 ± 2.74 ^ab^	6.31 ± 0.32	0.18 ± 0.07	14.21 ± 1.14 ^ab^	0.26 ± 0.06	0.71 ± 0.09
*p*-value						
Linear trend	0.305	0.162	0.473	0.089	0.957	0.068
Quadratic trend	0.008	0.979	0.997	0.380	0.946	0.243

Note: Superscripts of different letters (a and b) in the same column indicate significant differences between groups (*p* < 0.05).

## Data Availability

The authors confirm that the data supporting the findings of this study are available within the manuscript, tables, and figures.
